# Linking the maximum reported life span to the aging rate in wild birds

**DOI:** 10.1002/ece3.7471

**Published:** 2021-03-24

**Authors:** Canwei Xia, Anders Pape Møller

**Affiliations:** ^1^ Ministry of Education Key Laboratory for Biodiversity Science and Ecological Engineering College of Life Sciences Beijing Normal University Beijing China; ^2^ Ecologie Systématique Evolution Université Paris‐Sud CNRS AgroParisTech Université Paris‐Saclay Orsay Cedex France

**Keywords:** aging, bird, life span, mortality, Weibull distribution

## Abstract

Dozens of surrogates have been used to reflect the rate of aging in comparative biology. For wild organisms, the maximum reported life span is often considered a key metric. However, the connection between the maximum reported life span for a single individual and the aging rate of that species is far from clear. Our objective was to identify a pragmatic solution to calculate the aging rate from the maximum reported life span of wild birds. We explicitly linked the maximum reported life span to the aging process by employing a Weibull distribution and calculating the shape parameter in this model, which reflects the change in mortality across ages and be used as a surrogate for the aging rate. From simulated data, we demonstrated that the percentile estimator is suitable for calculating the aging rate based on the maximum reported life span. We also calculated the aging rate in 246 bird species based on published information from EURING and tested its relationship with body mass. Our study constitutes a new approach for using maximum reported life span in aging research. The aging rate calculated in the study is based on numerous assumptions/prerequisites and can be improved as more is learned about these assumptions/prerequisites.

## Graphical abstract

We explicitly linked the maximum reported life span to the aging process by employing a Weibull distribution and calculating the shape parameter in the model for 246 bird species based on published information from EURING.

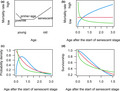

## 1 INTRODUCTION

To better understand the influence of aging, numerous studies have been conducted in comparative biology (e.g., Galvan & Møller, 2018; Lemaitre et al., 2020; Peron et al., 2019). Dozens of surrogates are used to reflect the rate of aging in these studies, and these surrogates can generally be grouped into three categories: sample statistics of life span, parameters in aging models, and maximum reported life span. When a sample of individuals was observed, the statistics (e.g., mean, median, and quantiles) of their longevities could be calculated as surrogates of the aging rate (Deevey, 1947; Lemaitre et al., 2020). Among these statistics, the 90% quantile of longevity was most frequently recommended (Peron et al., 2019; Wang et al., 2004), as it closely links the mortality of older individuals in a sample. The second category of surrogates concerns the fitting of a mathematical function to the relationship between age and rate of mortality (or proportion of surviving individuals), and the parameters in the model are used to describe the aging rate (Peron et al., 2019; Pinder et al., 1978). The Weibull model and the Gompertz model are two widely employed aging models, and the shape parameter in these two models exhibits the change in mortality through age (Ricklefs & Scheuerlein, 2002). To reflect the mortality difference at various life stages, the combined aging models can be used, for example, as three parts in the Siler model describing the mortality at the juvenile, prime‐age, and senescent stages (Siler, 1979). The performance of the above categories of surrogates is largely dependent on an adequate sample size. Therefore, these surrogates are commonly used in zoo animals and laboratory organisms to estimate the aging rate (Peron et al., 2019; Ricklefs & Scheuerlein, 2001), rather than wildlife in the field.

For wild organisms, species' maximum reported life spans are often taken as the key metric to compare aging rate among species (Tacutu et al., 2013; Thorley, 2020; Wasser & Sherman, 2010). The logic underlying this surrogate is that the lower the aging processes, the longer the recorded life span. The maximum reported life span can be easily obtained from online databases, such as the longest life span for animals in AnAge (https://genomics.senescence.info/species/) and the two oldest individuals for each bird species in EURING (https://euring.org/data‐and‐codes/euring‐databank). However, the maximum reported life span always increases with sample size (Møller, 2006; Xia & Møller, 2018). To control for the sampling effort, the adjusted maximum life span (e.g., the residual in the linear regression for maximum life span plotted against sample size) was used to reflect the aging rate. The maximum life span is also adjusted by the annual survival rate and/or body size in research, as there are positive correlations among these variables (Møller, 2006). Although various kinds of adjusted maximum life spans have been used in studies, the connections between these metrics from a few individuals and the aging rate of that species are far from clear (Gao et al., 2008; Wang et al., 2004). Another defect concerning this surrogate is that the maximum reported life span is not the real life span in many species (Moorad et al., 2012). For example, longevity records in EURING reflect the age of individuals when they are last seen or caught, with the remaining life span still unknown.

In this study, our aim was to find the link between the maximum reported life span and the aging rate of wild birds using information from EURING. The European Longevity Records offered by EURING is one of the two most widely used datasets on bird life span. Another dataset is offered by AnAge (Tacutu et al., 2013), which contains the maximum life span of birds all over the world, rather than only the European birds in EURING. However, there is more information for each species in EURING than in AnAge: For each species, EURING provides the life span of the two oldest individuals and the number of ringing recoveries (i.e., sampling effort), while the life span of one of the oldest individuals can be found in AnAge, with no clue about sampling effort. This extra information in EURING is crucial for revealing the aging rate.

**FIGURE 1:**
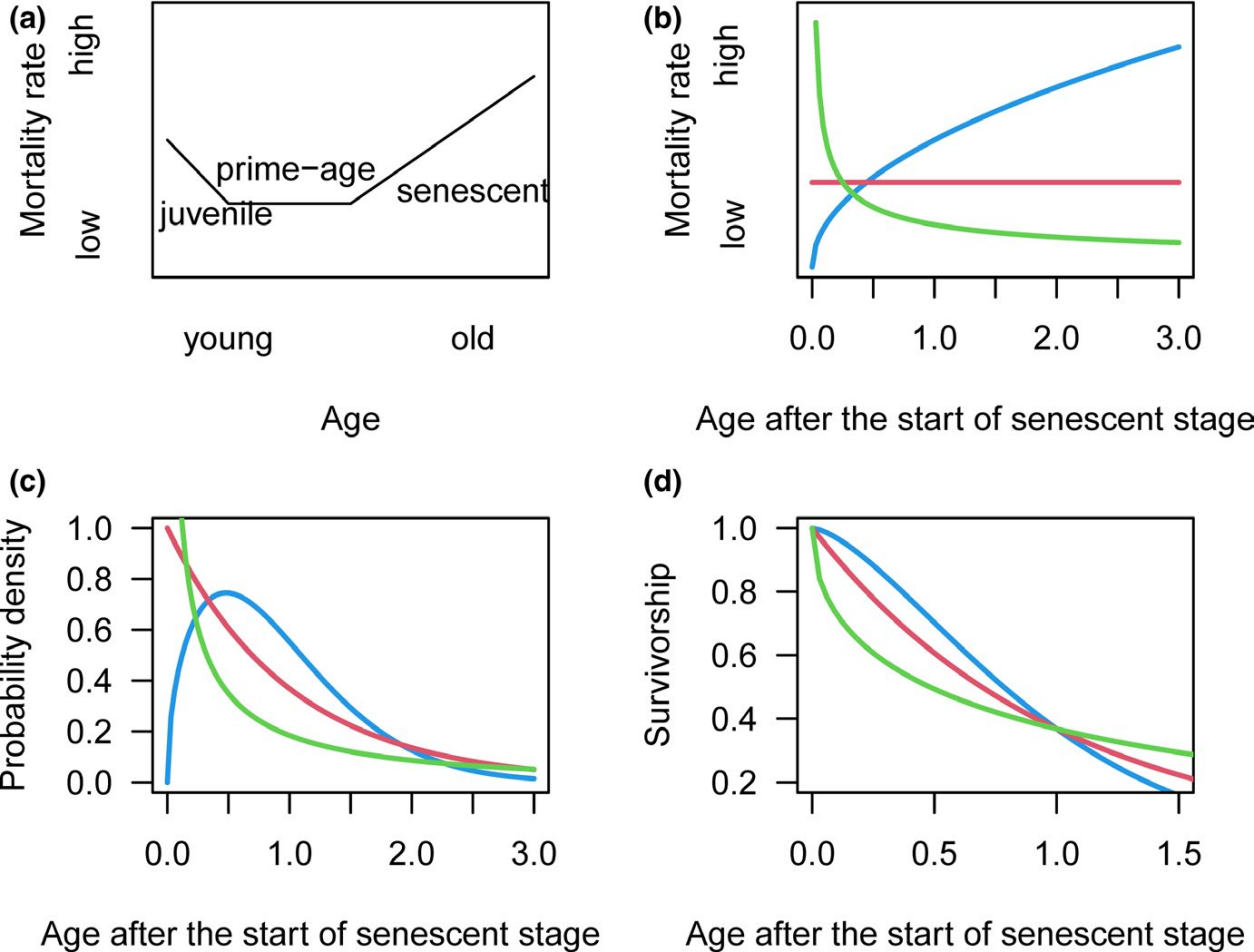
A hypothetical mortality rate plotted against age showing a change in mortality rate at different stages (a); the Weibull model of the aging process in mortality plotted against age (b), probability density of life span (c), and survivorship plotted against age (d). In (b), (c), and (d), location parameter “*a*” is 0; scale parameter “*b*” is 1; and shape parameter “*c*” is 1.5, 1, and 0.5 for the blue, red, and green lines, respectively

To reveal the aging rate, we must explicitly define “aging.” Here, we adopted the definition of “aging” as the change in mortality rate in the senescent stage (Baudisch, 2011). Generally, aging is a decline in physiological function with age, which can be reflected by the change in mortality rate (Kirkwood, 1977). A hypothetical mortality rate plotted against age is shown in Figure 1a, with the mortality rate being roughly classified into three stages: a decline in mortality rate at the juvenile stage, a low and constant mortality rate in prime age, and an increasing mortality rate at the senescent stage (Peron et al., 2019; Siler, 1979). Theoretically, the mortality rate toward the end of the life span can increase, decline, or remain constant, and these changes in mortality rate correspond to type I, type II, and type III survivorship curves (Deevey, 1947; Pearl, 1928).

We used the Weibull model to describe the change in mortality rate with age. The Weibull model provides a close approximation of the distribution of the lifetime for an object consisting of many parts in which death occurs when any of its parts fail (Collett, 2015; Rinne, 2009; Sharif & Islam, 1980). This model is widely used in life phenomena, from unicellular organisms (*Saccharomyces cerevisiae*, Liu & Acar, 2018; Guven et al., 2019) to fungi (*Penicillium bilaiae*, Friesen et al., 2006), plants (*Lemna gibba*, Chmilar & Laird, 2019), and animals (*Rhodnius neglectus*, Rabinovich et al., 2010; *Tribolium confusum*, Tanaka et al., 2016; Lepidoptera, Carroll & Sherratt, 2017; tyrannosaurs, Ricklefs, 2007; birds and mammals, Pinder et al., 1978; Ricklefs & Scheuerlein, 2002; *Homo sapiens*, Gurven & Fenelon, 2009; Hawkes et al., 2012). In the model, the change in mortality rate (*m*) is a function of age (*x*):

(1) $$m\left(x\right)=m_{0}+\frac{c}{b}*{\left(\frac{x‐a}{b}\right)}^{c‐1}$$

In Equation (1), “*m*_0_” is the mortality rate experienced by young adults of prime age; “*a*” is a location parameter that reflects the start age of the senescent stage; “*b*” is a scale parameter that influences the mean and median life span after the senescent stage; and “*c*” is a shape parameter that controls the direction and rate of change in the age‐specific mortality rate (Figure 1b). As “*m*_0_” only influences the intercept, rather than the change in mortality rate throughout the life span, we omitted “*m*_0_” and simplified the equation:

(2) $$m_{X}\left(x\right)=\frac{c}{b}*{\left(\frac{x‐a}{b}\right)}^{c‐1}$$

The corresponding probability density function of life span (Figure 1c) is

(3) $$f_{X}\left(x\right)=m_{X}\left(x\right)*\exp\left[‐\int_{a}^{x}m_{X}\left(x\right)\cdot dx\right]=\frac{c}{b}*{\left(\frac{x‐a}{b}\right)}^{c‐1}*\exp\left[‐{\left(\frac{x‐a}{b}\right)}^{c}\right]$$

and the survivorship against age (Figure 1d) is

(4) $$s_{X}\left(x\right)=\exp\left[‐\int_{a}^{x}m_{X}\left(x\right)\cdot dx\right]=\exp\left[‐{\left(\frac{x‐a}{b}\right)}^{c}\right]$$

In this study, by using the framework of the Weibull model, we calculated the aging rate in wild birds based on published information (for each species, the maximum reported life span of the two oldest individuals and the number of ringing recoveries) from EURING and tested its accuracy with simulation data. We also tested the relationship between aging rate and body mass as an example showing how to use the aging rate calculated in the study. Finally, we fully discussed the assumption/prerequisite of this work and analyzed the potential biases in the calculation of the aging rate.

## 2 MATERIALS AND METHODS

### 2.1 Data source

We obtained information about the maximum reported life span and number of ringing recoveries from EURING. For each species, the life span of the two oldest individuals among ringed European birds was reported. The last version of this dataset was updated on 5 April 2017. The number of ringing recoveries was last updated in approximately 2015. For these species, we extracted information on the mean body mass of adult birds from Dunning (2008) and the age at first reproduction from Møller (2006). In total, we obtained the above information from 246 bird species. The dataset used in this study is reported in Appendix S1.

### 2.2 Calculate aging rate based on real life span

Among the dozens of parameter estimators in the Weibull distribution, we chose the percentile estimator developed by Dubey (1967), which can calculate shape parameter “*c*” based on at least two observations. Here, we briefly introduced this method.

The cumulative distribution function for the Weibull model is

(5) $$F_{X}\left(x\right)=\int_{a}^{x}f_{X}\left(x\right)=1‐\exp\left[‐{\left(\frac{x‐a}{b}\right)}^{c}\right]$$

When we know the value of an observation ($x_{p}$) and its corresponding percentile (p), by solving $F_{X}\left(x_{p}\right)=p$, we obtain

(6) $$Ln\left[‐Ln\left(1‐p\right)\right]=c*\left[Ln\left(x_{p}‐a\right)‐Ln\left(b\right)\right]$$

When we know the values of two observations ($x_{p1}<x_{p2}$) and their corresponding percentiles (0<p1<p2<1), we obtain

(7) $$\hat{c}=\frac{Ln\left[‐Ln\left(1‐p1\right)\right]‐Ln\left[‐Ln\left(1‐p2\right)\right]}{Ln\left(x_{p1}‐a\right)‐Ln\left(x_{p2}‐a\right)}$$

where “$\hat{c}$” indicates the estimate of parameter “*c*.” Using Mosteller's theorem (Mosteller, 1946) and Rao's lemma (Rao, 1952), we know that “$\hat{c}$” is asymptotically normal with variance:

(8) $$Var\left(\hat{c}\right)=\frac{\left(\frac{q1}{k1^{2}}+\frac{q2}{k2^{2}}‐2*\frac{q1}{k1*k2}\right)}{n*{\left(Ln\left(x_{p1}‐a\right)‐Ln\left(x_{p2}‐a\right)\right)}^{2}}$$

where *n* is the total number of observations (i.e., sample size); $q1=\frac{p1}{1‐p1}$ and $q2=\frac{p2}{1‐p2}$; and $k1=‐Ln\left(1‐p1\right)$ and $k2=‐Ln\left(1‐p2\right)$. As “$\hat{c}$” is asymptotically normal, we can construct a 95% confidence interval for “*c*” as

(9) $$\left(\hat{c}‐1.96*Sd\left(\hat{c}\right),\hat{c}+1.96*Sd\left(\hat{c}\right)\right)$$

where $Sd\left(\hat{c}\right)$ is the standard deviation and is equal to $\sqrt{Var\left(\hat{c}\right)}$.

From the above equations, we can calculate the aging rate (i.e., the point estimation of shape parameter “*c*”) and its variation (variance, standard deviation, and confidence interval) based on the sample size, values of two observations, and location parameter “*a*.” Without having separate data to estimate the start age of the senescent stage (i.e., location parameter “*a*”), it is suggested that the age at first reproduction be used as the start age of the senescent stage (Pinder et al., 1978; Wrycza et al., 2015). The logic underlying this approach is that functional deterioration always arises after sexual maturation, as natural selection that rules out defective genes is less effective after reproduction than before reproduction (Kirkwood, 1977).

### 2.3 Calculate aging rate based on reported life span

Now, we must face the real challenge in that we only know the reported life span, that is, the age of the individuals when they are last seen or caught, with the remaining life span after that being unknown. As the probability of finding a particular individual in the field is low (e.g., less than 1% of banded birds tend to be recovered (Cleminson & Nebel, 2012)), we assumed that a banded individual can only be found once at most after banding. We also assume that the time when a particular individual can be found belongs to a uniform distribution, and the domain is from the start of the senescent stage to its real life span when we only focus on individuals with a reported life span larger than the start age of the senescent stage. Based on the prerequisites, given that the real life span is *x*, the function of the conditional probability density of the reported life span (*y*) is

(10) $$f_{Y|X}\left(y|x\right)=\frac{1}{x‐a},\;\text{for}\;a\leq y\leq x$$

Equation (10) indicates that an individual who died at age (*x*) could be observed before age (*x*) but could not be observed after age (*x*). Combining Equation (3) and Equation (10), we can obtain the probability density function for the reported life span (*y*):

(11) $$f_{Y}\left(y\right)=\int_{y}^{\infty }f_{Y|X}\left(y|x\right)*f_{X}\left(x\right)\cdot dx=\int_{y}^{\infty }\left\{\frac{c}{b^{c}}*{\left(x‐a\right)}^{\left(c‐2\right)}*\exp\left[‐{\left(\frac{x‐a}{b}\right)}^{c}\right]\right\}\cdot dx$$

Equation (11) implies that the probability of a reported life span at age (*y*) is attributed to individuals with a real life span (*x*) equal to or larger than age *y*.

There is no elementary function for $f_{Y}\left(y\right)$ according to the Risch algorithm (Chiccoli et al., 1990). Therefore, we do not directly estimate parameter “*c*” from $f_{Y}\left(y\right)$. Our pragmatic solution to this problem was to treat the reported life span as the real life span and calculate parameter “*c*” based on the percentile estimators developed by Dubey (1967). We used the simulation data to test whether this pragmatic solution is feasible. For simulation data, we tested the linear relationship between real parameter “*c*” (which we set in simulation) and estimate “$\hat{c}$” and calculated the probability of the 95% confidence interval containing the real value of “*c*.” If “$\hat{c}$” is linearly related to “*c*” and the 95% confidence interval has a high probability of containing the real value of “*c,*” parameter “*c*” can be reflected by estimate “$\hat{c}.$” We also tested the bias concerning estimate “$\hat{c},$” that is, whether the deviation between “$\hat{c}$” and “*c*” is related to the value of “*c,*” parameter “*b,*” and sample size. In this way, we can know the constraints of this pragmatic solution.

### 2.4 Simulation data

The following steps were employed to generate simulation data. First, we generated a real life span sample from the Weibull distribution, in which the sample size was obtained from a uniform distribution with a domain range of 100–100,000, location parameter “*a*” was 0, scale parameter “*b*” was obtained from a uniform distribution with a domain range of 1–15, and the shape parameter was obtained from a uniform distribution with a domain range of 0.03–10. As parameter “*a*” equals 0, the life span of the sample is the life span after the start of the senescent stage. The range of parameters in the simulation data is sufficient to include estimated parameters in previous research on the aging of birds (e.g., Pinder et al., 1978; Ricklefs & Scheuerlein, 2001). Second, we obtained the sample of reported life spans from the sample of real life spans. For each individual in the sample, the reported life span is from a uniform distribution with a domain range of 0 to the real life span of this individual. Third, we chose the largest and the second largest values from the sample of reported life spans, which correspond to the maximum reported life span of the two oldest individuals in the sample, and used these two values, their percentile, and sample size to calculate “$\hat{c}$” and its 95% confidence interval from Equations (7), (8), (9). The percentiles for the largest and the second largest reported life spans are $\frac{n}{n+1}$ and $\frac{n‐1}{n+1}$, respectively, where “*n*” is the sample size.

We repeated the above three steps 100,000 times to obtain 100,000 samples and calculated “$\hat{c}$” and its 95% confidence interval for each sample. Then, we tested the linear relationship between estimate “$\hat{c}$” and the real value of parameter “*c*” and calculated the probability of a 95% confidence interval containing the real value of “*c*.”

### 2.5 Aging rate against body mass

After confirming that percentile estimators developed by Dubey (1967) are applicable for the two maximum reported life spans from simulation data, we used these estimators to calculate the aging rate (i.e., “$\hat{c}$”) and its standard deviation for wild birds. As suggested by previous research (Pinder et al., 1978; Wrycza et al., 2015), we used the age at first reproduction as the start age of the senescent stage (i.e., location parameter “*a*”). The life span of the two oldest individuals and the number of ringing recoveries (i.e., sample size “*n*”) from 246 bird species were downloaded from EURING. The percentiles for the largest and the second largest reported life spans are $\frac{n}{n+1}$ and $\frac{n‐1}{n+1}$, respectively. For 9 species, the largest and second largest reported life spans were equal because the life span records in EURING accounted for 1 month. For this situation, we added 10 days to the longest reported life span, which is the expected value of the difference between the longest reported life span and the second longest reported life span in this situation.

After calculating the aging rate (i.e., “$\hat{c}$”) from Equation (7) and its variation from Equation (8), we tested the relationship between aging rate and body mass as an example showing how to use these data. From Equation (8), we can see that the variance of estimate “$\hat{c}$” is related to sample size “*n*” (i.e., number of ringing recoveries). As the number of ringing recoveries is highly unbalanced among species, ranging from 3 to 375,858, the variance in aging rate is also very different among species. By intuition, we should trust the point estimation with small variation. Therefore, we used weighted linear regression, with more weight given to the species with smaller standard deviations. Aging rate and body mass were *Ln*‐transformed, and then, they were used in regression. The standard deviation for the aging rate was transformed according to the first‐order Taylor expansion (Canchola et al., 2017): $Sd\left(Ln\left(\hat{c}\right)\right)\approx Sd\left(\hat{c}\right)/\hat{c}$.

All analyses were conducted using R 4.0.0. Weighted regression was performed using the “metafor” package (Viechtbauer, 2010). Data were reported as the mean ± standard error. The results were considered significant if *p* < .05 (two‐tailed test).

## 3 RESULTS

**FIGURE 2:**
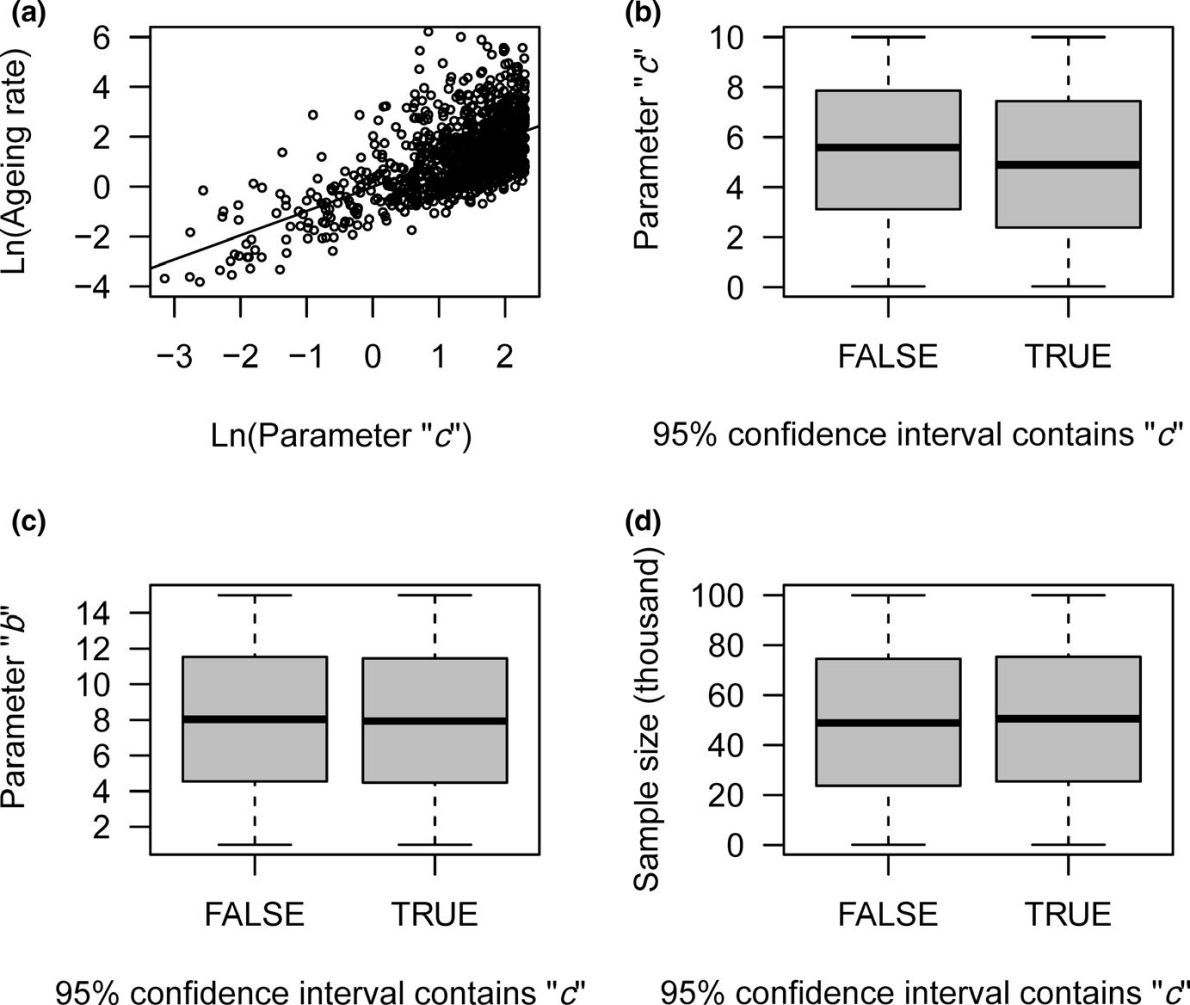
Aging rate (i.e., estimate “$\hat{c}$”) is strongly related to the real value of parameter “*c*” (a); the difference in the values of parameter “*c*” (b), parameter “*b*” (c), and sample size (d) of the two groups divided by whether the 95% confidence interval of estimate “$\hat{c}$” contains the real value of parameter “*c*.” To avoid overlap, only 1000 randomly selected data points are shown in (a)

In this study, we used the Weibull distribution to model the change in mortality rate in the senescent stage and calculated estimate “$\hat{c}$” (i.e., aging rate) from the percentile estimator. Based on the simulation data, estimate “$\hat{c}$” is significantly linearly related to the real value of parameter “*c*” (Figure 2a), with a coefficient of 0.913 ± 0.004 (*t*_99998_ = 219.05, *p* < .001).

The 95% confidence interval, calculated by the percentile estimator, has an 80.4% probability of containing the real value of “*c*.” We divided the simulation data into two groups based on whether the 95% confidence interval contains the real value of “*c*.” The effect size of the standardized mean difference between these two groups is −0.175 for the real value of “*c*” (Figure 2b), −0.017 for parameter “*b*” (Figure 2c), and 0.040 for sample size (Figure 2d).

**FIGURE 3:**
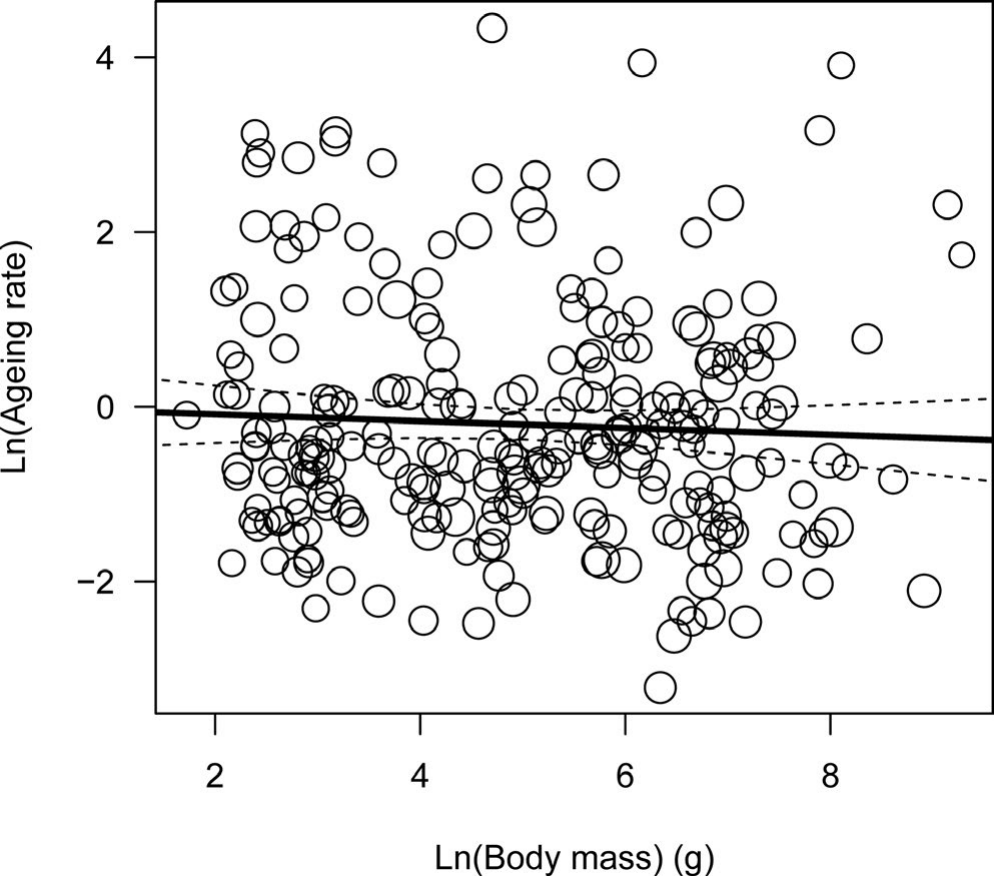
Relationship between aging rate and body mass. The size of the circles indicates the relative weight: The larger a circle is, the smaller the standard deviation; the actual line indicates the fitted line of the weighted regression; and the dotted line indicates the 95% confidence interval of the fitted line

For the 246 bird species, the aging rate reflected by estimate “$\hat{c}$” was 2.79 ± 0.49. The point estimation of the aging rate and its standard deviation for each species are reported in Appendix S1. The aging rate slightly decreased as the body mass increased (Figure 3). However, the coefficient −0.039 ± 0.049 was not significantly different from 0 (*z* = −0.798, *p* = .425).

## 4 DISCUSSION

Species maximum reported life span, or adjusted maximum reported life span, is often used as the key metric to compare the aging rates of wild species (Tacutu et al., 2013; Wasser & Sherman, 2010). However, the connection between the life span of a single individual and the aging rate of the species is far from clear (Gao et al., 2008; Wang et al., 2004). In this study, we explicitly link the maximum reported life span to the aging process by employing the Weibull model and calculating shape parameter “*c*” in the model, which reflects the change in mortality through age, as a surrogate of the aging rate.

Whether the reported life span can reflect the aging rate is an open question, as the remaining life span after the reported life span is unknown (Moorad et al., 2012). In this study, we deduced the probability density function of the reported life span. As there is no elementary function for this probability density function (Chiccoli et al., 1990), we do not directly estimate parameter “*c*” (i.e., aging rate) from this probability density function. From the simulation data, we demonstrated that the percentile estimator developed by Dubey (1967) is suitable for estimating “$\hat{c}$” based on the maximum reported life span. Estimate “$\hat{c}$” is significantly linearly related to the real value of parameter “*c,*” and the coefficient, 0.913, is very close to 1. The 95% confidence interval contains the real value of “*c*” with an 80.4% probability. Except for the real value of “*c,*” the differences in the parameter “*b*” and sample size of the two groups divided by whether the 95% confidence interval contains the real value of “*c*” are smaller than the small effect size of 0.10 suggested by Cohen (1988). These results imply that there is no obvious constraint for estimating “$\hat{c}$” using the maximum reported life span from the percentile estimator.

A significant linear relationship between the maximum reported life span and body mass was repeatedly observed in bird species (e.g. Møller, 2009; Xia & Møller, 2018). However, this linear relationship was not observed in this study when the aging rate, rather than the maximum reported life span, was used. Similar to this result, Peron et al. (2019) showed that aging rate and maximum life span are affected by different life history features; Lemaitre et al. (2020) found that median life span in female mammals is longer than that of conspecific males, while there is no consistent sex difference in aging rates. These contradictions reflect that the aging rate and life span are not equivalent (Moorad et al., 2012; Peron et al., 2019) and suggest that the aging rate, rather than the maximum life span, should be used in comparative biology if the aim is to understand the influence of aging (Lemaitre et al., 2020; Peron et al., 2019).

This study is based on the framework of the Weibull model. We admit that the Weibull model fitting the aging process is the assumption/prerequisite of our analysis, and we do not test whether this assumption/prerequisite is suitable for the 246 bird species we analyzed. Although numerous research projects have demonstrated that the Weibull model is an adequate model for the aging process in many organisms, including birds (Pinder et al., 1978; Ricklefs, 2010; Ricklefs & Scheuerlein, 2002), we do not take it for granted and suggest that this assumption/prerequisite is open for empirical testing. Another assumption/prerequisite is that the study considers the start age of the senescent stage (i.e., location parameter “*a*” in the model). We used the age at first reproduction as the start age of the senescent stage. This approach is recommended by previous research when there is insufficient information for determining the start age of the senescent stage (Pinder et al., 1978; Wrycza et al., 2015). This value should be revised if the real start age of the senescent stage becomes known.

Mortality in the senescent stage is composed of two components: constant mortality “*m*_0_” and the change in mortality through aging (Equation 1). Previous research found that constant mortality “*m*_0_” is relatively small compared with the change in mortality through aging (Gurven & Fenelon, 2009; Hawkes et al., 2012; Ricklefs, 2007; Ricklefs & Cadena, 2008). As there are no data in the study to estimate “*m*_0_” (i.e., the mortality in the prime‐age stage) for the birds we analyzed, we omitted this mortality in the analysis. When “*m*_0_” is included, the probability density function of the life span is

(12) $$f_{X}\left(x\right)=m_{X}\left(x\right)*\exp\left[‐\int_{a}^{x}m_{X}\left(x\right)\cdot dx\right]=\left[m_{0}+\frac{c}{b}*{\left(\frac{x‐a}{b}\right)}^{c‐1}\right]*\exp\left[‐m_{0}*x‐{\left(\frac{x‐a}{b}\right)}^{c}\right]$$

The probability that an individual has a relatively long life span is lower based on Equation (12) than based on Equation (3). Correspondingly, the difference between two extreme life spans also decreases as a positive “*m*_0_” is included. In that way, estimate “$\hat{c}$” is influenced by the value of “*m*_0_.” Therefore, to alleviate this bias, we suggest including extra information to estimate the value of “*m*_0_.”

There is another potential bias concerning the calculated aging rate in wild birds. For the 246 bird species, we used the number of ringing recoveries as sample size “*n*.” This approach implies that the life spans of all individuals belong to the same distribution. As pointed out by Siler (1979) and adopted by subsequent research (e.gLemaitre et al., 2020; Peron et al., 2019), different distributions are used to describe the life spans of the individuals who died in different stages (i.e., juvenile, prime‐age, and senescent stages). Clearly, the sample size for the senescent stage is less than the number of ringing recoveries. From Equation (7), we can obtain

(13) $$\hat{c}\propto \left\{Ln\left[Ln\left(\frac{n+1}{2}\right)\right]‐Ln\left[Ln\left(n+1\right)\right]\right\}$$

This is an increasing function for sample size “*n*.” Therefore, estimated value of parameter “*c*” should be changed when sample size “*n*” is overestimated. This bias can be removed if we use the correct sample size by focusing on the individuals alive in the senescent stage.

## 5 CONCLUSION

We described a pragmatic solution for calculating the aging rate from the maximum reported life span of wild birds and tested the accuracy of this approach by using simulated data. Although defects in the maximum reported life span have been repeatedly reported, it is still the crucial information, which can be easily collected, for most wild birds. Our study provides a new approach for using this information. The aging rate calculated in the study is based on numerous assumptions/prerequisites and can be improved when we know more details of these assumptions/prerequisites.

## CONFLICT OF INTEREST

The authors declare no conflict of interest.

## AUTHOR CONTRIBUTIONS

**Canwei Xia:** Conceptualization (supporting); data curation (lead); formal analysis (lead); funding acquisition (equal); investigation (equal); methodology (equal); project administration (supporting); resources (equal); software (equal); supervision (supporting); validation (equal); visualization (equal); writing‐original draft (equal); writing‐review & editing (equal). **Anders Pape Møller:** Conceptualization (lead); data curation (supporting); formal analysis (supporting); funding acquisition (equal); investigation (equal); methodology (equal); project administration (lead); resources (equal); software (equal); supervision (lead); validation (equal); visualization (equal); writing‐original draft (equal); writing‐review & editing (equal).

## Supporting information

Appendix S1

## Data Availability

The dataset used in this study is included in Appendix S1.
